# Chest wall loading during supine and prone position in patients with COVID-19 ARDS: effects on respiratory mechanics and gas exchange

**DOI:** 10.1186/s13054-022-04141-7

**Published:** 2022-09-13

**Authors:** Michele Umbrello, Sergio Lassola, Andrea Sanna, Rocco Pace, Sandra Magnoni, Sara Miori

**Affiliations:** 1 SC Anestesia e Rianimazione II, Ospedale San Carlo Borromeo, ASST Santi Paolo e Carlo – Polo Universitario, Milan, Italy; 2 Department of Anesthesia and Intensive Care, Santa Chiara Hospital, Trento, Italy

**Keywords:** COVID-19, ARDS, Chest wall compression, Prone positioning

## Abstract

**Background:**

Recent reports of patients with severe, late-stage COVID-19 ARDS with reduced respiratory system compliance described paradoxical decreases in plateau pressure and increases in respiratory system compliance in response to anterior chest wall loading. We aimed to assess the effect of chest wall loading during supine and prone position in ill patients with COVID-19-related ARDS and to investigate the effect of a low or normal baseline respiratory system compliance on the findings.

**Methods:**

This is a single-center, prospective, cohort study in the intensive care unit of a COVID-19 referral center. Consecutive mechanically ventilated, critically ill patients with COVID-19-related ARDS were enrolled and classified as higher (≥ 40 ml/cmH_2_O) or lower respiratory system compliance (< 40 ml/cmH_2_O). The study included four steps, each lasting 6 h: Step 1, supine position, Step 2, 10-kg continuous chest wall compression (supine + weight), Step 3, prone position, Step 4, 10-kg continuous chest wall compression (prone + weight). The mechanical properties of the respiratory system, gas exchange and alveolar dead space were measured at the end of each step.

**Results:**

Totally, 40 patients were enrolled. In the whole cohort, neither oxygenation nor respiratory system compliance changed between supine and supine + weight; both increased during prone positioning and were unaffected by chest wall loading in the prone position. Alveolar dead space was unchanged during all the steps. In 16 patients with reduced compliance, PaO_2_/FiO_2_ significantly increased from supine to supine + weight and further with prone and prone + weight (107 ± 15.4 vs. 120 ± 18.5 vs. 146 ± 27.0 vs. 159 ± 30.4, respectively; *p* < 0.001); alveolar dead space decreased from both supine and prone position after chest wall loading, and respiratory system compliance significantly increased from supine to supine + weight and from prone to prone + weight (23.9 ± 3.5 vs. 30.9 ± 5.7 and 31.1 ± 5.7 vs. 37.8 ± 8.7 ml/cmH_2_O, *p* < 0.001). The improvement was higher the lower the baseline compliance.

**Conclusions:**

Unlike prone positioning, chest wall loading had no effects on respiratory system compliance, gas exchange or alveolar dead space in an unselected cohort of critically ill patients with C-ARDS. Only patients with a low respiratory system compliance experienced an improvement, with a higher response the lower the baseline compliance.

**Supplementary Information:**

The online version contains supplementary material available at 10.1186/s13054-022-04141-7.

## Background

Coronavirus disease 2019 (COVID-19) is a viral infectious disease caused by a novel coronavirus (SARS-CoV-2). It primarily affects the respiratory system causing mild to severe respiratory illness, possibly leading to hypoxemic respiratory failure requiring invasive mechanical ventilation and ICU admission [1]. In a small but significant proportion of patients, conventional lung protective ventilation is not sufficient to relieve hypoxemia and prevent ventilator-induced lung injury, and other strategies should be taken into account.

Prone positioning has been used for over 30 years in the management of patients with acute respiratory distress syndrome (ARDS). This maneuver has consistently proven capable of improving oxygenation in patients with acute respiratory failure. Several mechanisms can explain this observation, including possible intervening net recruitment and more homogeneously distributed alveolar inflation, as well as more homogeneous distribution of perfusion and better V/Q matching, independent of extent of recruitment [2, 3]. It is also progressively becoming clear that prone positioning may reduce the non-physiological stress and strain associated with mechanical ventilation, thus decreasing the risk of ventilator-induced lung injury, which is known to adversely impact patient survival [4].

As exemplified by prone positioning, regional variations in lung and chest wall properties provide possibilities for modifying transpulmonary pressures [5–7] and suggest that clinical application of external pressure on the chest wall may be a useful approach to lung protection [8]. Loading of the chest wall reduces chest wall compliance, and an increased driving pressure (or a reduced respiratory system compliance) is expected if PEEP and tidal volume are unchanged [9], provided that lung compliance does not simultaneously increase. While such mechanic improvement, due to an increased caudal expansion of the lungs, was previously demonstrated [10], application of chest wall loading was seldom performed in the clinical practice. Indeed, recent reports of patients with severe late-stage ARDS caused by COVID-19 (C-ARDS) with reduced respiratory system compliance described so-called “paradoxical” decreases in plateau pressure and increases in respiratory system compliance [11–16] after chest wall compressions in the supine position, and renewed the interest on this maneuver. However, no data are available as to the effect of chest wall loading in patients with less reduced respiratory system compliance or in an earlier phase of the disease.

The aim of the present investigation was to assess the effect of chest wall loading during supine and prone position in an unselected cohort of critically ill patient with C-ARDS. Our main hypothesis was that chest wall loading increases respiratory system compliance in all patients with C-ARDS. Secondary aims were to compare the effects of loading the chest wall between patients with normal or reduced respiratory system compliance, to assess whether the response to chest wall loading during supine position is able to predict the oxygenation or respiratory mechanics response during prone position and to evaluate the effect of chest wall loading in an early or a late phase of C-ARDS.

## Methods

We conducted a prospective, observational clinical investigation to assess the effect of chest wall compression by sand bags (10 kg) on gas exchange and mechanical properties of the respiratory system.

The research was performed in accordance with the Declaration of Helsinki. Ethical approval for this study (Rep. Int. 1795) was provided by the Comitato Etico per le Sperimentazioni Cliniche of the Azienda Provinciale per i Servizi Sanitari di Trento (Chairperson dott. Giuseppe Moretto); written informed consent was obtained according to Italian regulations.

### Subjects

All subjects aged ≥ 18 years admitted from November 2020 to May 2021 to the general ICU of a tertiary care hospital for acute respiratory distress syndrome and with confirmed SARS-CoV-2 infection were consecutively enrolled. Confirmed infection was defined as a positive reverse transcriptase polymerase chain reaction from a nasopharyngeal swab, associated with symptoms, signs and radiological findings suggestive of COVID-19. ARDS was defined following the Berlin definition [17]. All subjects were deeply sedated and mechanically ventilated at enrolment. The clinical management of subjects was standardized according to local and regional suggestion [18].

Exclusion criteria were: age less than 18 years old, pregnancy, severe hemodynamic instability, any factors that contraindicate the application of chest wall weights (rib fractures, burns, severe chest or abdominal deformities).

### Data collection

Data on demographic characteristics (age, BMI, gender), clinical features, medical history, time from onset of symptoms to hospital and ICU admission, time from ICU admission to intubation, ICU and hospital length of stay and ICU outcome were all recorded. SAPS II at ICU admission and SOFA score at the day of enrollment were calculated.

### Protocol

All patients were treated according to local clinical practices and following national recommendations [18]; in particular, before enrolment in the study, ventilatory parameters as FiO_2_, PEEP, tidal volume and respiratory rate, as well as sedation level were left to the clinicians' decisions and have not been modified during the observation. Positive end-expiratory pressure (PEEP) was set according to the best respiratory system compliance (Crs) assessed with a recruitment maneuver followed by a decremental PEEP trial [19].

Patients who were enrolled underwent four steps, each lasting 6 h: Step 1, supine position, Step 2, 10-kg continuous chest wall compression (supine + weight), Step 3, prone position, Step 4, 10-kg continuous chest wall compression (prone + weight). Additional file 1: Figure S1 shows a scheme of the study protocol. Chest wall compression was obtained through two sand bags, each weighing 5 kg. We arbitrarily chose a 10-kg weight because in a previous investigation the pressure exerted by this weight seemed to induce significant changes to the respiratory system [14]. The sandbags were distributed exclusively on the chest wall surface, over the sternum and ribs in the supine position and over the thoracic spine and shoulder blades in prone position; in particular, as we used two sandbags with a total weight of 10 kg, with an approximate contact surface of 15 × 20 cm, the pressure applied was calculated to be about 35 cmH_2_O. In no case did we apply any abdominal compression.

During the last hour of each step, the driving and airway plateau pressure were measured; we collected an arterial blood sample, recorded the EtCO_2_ and calculated derived gas exchange parameters as alveolar dead space and the mechanical properties of respiratory system. EtCO_2_ was measured with the mainstream capnometer of the mechanical ventilator. The compliance of the respiratory system was defined as the tidal volume divided by the inspiratory driving pressure. The ventilatory ratio, a measure of impaired ventilation [20], was defined as: [minute ventilation (ml/min) × PaCO_2_ (mmHg)]/(predicted body weight × 100 × 37.5), where higher values indicate a more impaired ventilation. Hemodynamic parameters were continuously monitored. To avoid any possible influence of the degree of trunk inclination on the results, the measurements were made with patients at 0 degrees in all four steps of the study [21].

If any side effect or sign of poor tolerance [22], such as pressure sores, endotracheal tube displacement, obstruction of the endotracheal tube, venous access loss, discomfort feeling, non-cooperation and aggravated cough was detected, the study was allowed to stop. In particular, pressure sores were prevented with the use of positioning dressings to help offload pressure points between weight and skin.

### Classifications

Patients were classified as having a higher or a lower respiratory system compliance based on the value in supine position, using a cutoff of 40 ml/cmH_2_O [23]; the phase of C-ARDS was classified as early if within the first week and late if beyond the first week of ICU admission [24]. Eventually, the analysis was performed on the baseline value of airway driving pressure, as a surrogate for lung strain [25].

### Statistics

Continuous variables are presented as mean ± standard deviation if normally distributed or medians (25th; 75th quartile) if not; categorical variables are shown as number and percentages. Subjects were divided in groups according to the respiratory system compliance in supine position at enrolment in the study (higher vs. lower compliance). Continuous variables were compared with appropriate parametric or nonparametric tests according to their distribution, and categorical variables were compared with chi-square tests.

The analysis of the variables during the 4 consecutive steps in the whole case-mix was performed by one-way analysis of variance, with appropriate post hoc tests. The comparison between patients with normal and reduced compliance across different steps of the study was performed by analysis of variance for repeated measurements, with the study step as a within-subject factor and the higher or lower compliance as a fixed, between-subject factor. The model included the interaction effect of the step on the respiratory system compliance. The statistical significance of the within-subject factors was corrected with the Greenhouse–Geisser method. Pairwise, post hoc multiple comparisons were carried out according to Tukey method.

Based on the data from a wide sample of critically ill, COVID-19 subjects enrolled in Italy, in which the average respiratory system compliance was 41 ± 14 ml/cmH_2_O [26], and hypothesizing that chest wall loading increases respiratory system compliance by 30%, as reported by a recent report [14], we calculated that a sample of 40 subjects would be required for an 80% power, at an alpha = 0.05. The statistical analysis was carried out with STATA version 14.0 (Statacorp, College Station, TX, USA); two-tailed *p* values < 0.05 were considered for statistical significance.

## Results

Forty consecutive patients were enrolled. The main characteristics at baseline, parameters at enrolment in the study and outcomes are shown in Table 1. Patients were studied after an average ICU stay of 10 [8; 11] days and after a period of invasive mechanical ventilation of 9 [6; 10] days (higher compliance 8 [5; 9] vs. lower compliance 10 [8; 11] days, *p* = 0.0087); 16 patients were enrolled in the early phase, whereas 24 in a late phase. We did not record any side effect or sign of poor tolerance after chest wall loading.

**Table 1:** Baseline characteristics of the whole cohort and comparison between patients with lower or higher respiratory system compliance at enrolment

	Whole cohort *N* = 40	Lower Crs *N* = 16	Higher Crs *N* = 24	*p* value
*Anthropometric measures*				
Male Sex (n-%)	30 (75)	10 (62.5)	20 (83.3)	0.1360
Weight (kg)	86.1 ± 17.8	79.1 ± 10.2	90.1 ± 20.3	0.0416
Height (m)	1.72 ± 0.08	1.68 ± 0.09	1.73 ± 0.07	0.0959
Predicted body weight (kg)	67.6 ± 7.6	65.1 ± 8.5	69.2 ± 6.7	0.1041
Body-mass index (kg/m^2^)	29.1 ± 5.6	27.9 ± 5.3	29.9 ± 5.7	0.2549
Age (years)	68.0 ± 8.9	71.8 ± 6.6	65.5 ± 9.6	0.0277
*Comorbidities*				
Hypertension (n-%)	29 (72.5)	11 (68.8)	18 (75.0)	0.6650
Diabetes (n-%)	5 (12.5)	1 (6.25)	4 (16.7)	0.9524
Renal failure (n-%)	3 (7.5)	1 (6.25)	2 (8.33)	0.0601
Cardiac failure (n-%)	3 (7.5)	1 (6.25)	2 (8.33)	0.0601
Respiratory disease (n-%)	6 (15.0)	2 (12.5)	4 (16.7)	0.1307
*ICU severity scores*				
SAPS II	37.1 ± 6.5	38.6 ± 6.2	36.2 ± 6.6	0.2583
PaO_2_/FiO_2_ at ICU admission (mmHg)	174 ± 87	163 ± 79	182 ± 93	0.5207
*Parameters at enrolment*				
Temperature (C°)	36.6 ± 0.2	36.5 ± 0.2	36.6 ± 0.2	0.4319
RASS	− 4 ± 0	− 4 ± 0	− 4 ± 0	> 0.999
FiO_2_	0.58 ± 0.08	0.64 ± 0.07	0.54 ± 0.06	< 0.0001
Tidal volume (mL)	488 ± 81	396 ± 38	550 ± 18	< 0.0001
Tidal volume/PBW (mL/kg)	7.2 ± 1.2	6.1 ± 1.1	8.0 ± 0.8	< 0.0001
Respiratory rate (1/min)	18.5 ± 3.76	22.6 ± 2.0	15.8 ± 1.2	< 0.0001
Minute ventilation (L/min)	8.76 ± 0.73	8.91 ± 0.83	8.66 ± 0.66	0.2883
PEEP (cmH_2_O)	9.3 ± 1.6	10.3 ± 1.9	8.8 ± 1.0	0.0024
*Outcome*				
ICU Non-survivors (n-%)	13 (32.5)	9 (56.3)	4 (16.7)	0.0090
Hospital length of stay (days)	32.0 ± 12.9	31.7 ± 15.0	32.1 ± 12.9	0.9182
ICU length of stay (days)	24.0 ± 11.3	26.2 ± 12.1	22.5 ± 10.8	0.3129

Crs: respiratory system compliance; ICU: intensive care unit; SAPS II: Simplified Acute Physiology Score 2^nd^ version; RASS: Richmond agitation sedation scale; PBW: predicted body weight; PEEP: positive end-expiratory pressure

The analysis on the variables was performed by unpaired Student’s t test for continuous data, or by Chi-square test for categorical data. Two-tailed *p* values < 0.05 were considered statistically significant

### Effects of chest wall loading—whole cohort

**Fig. 1:**
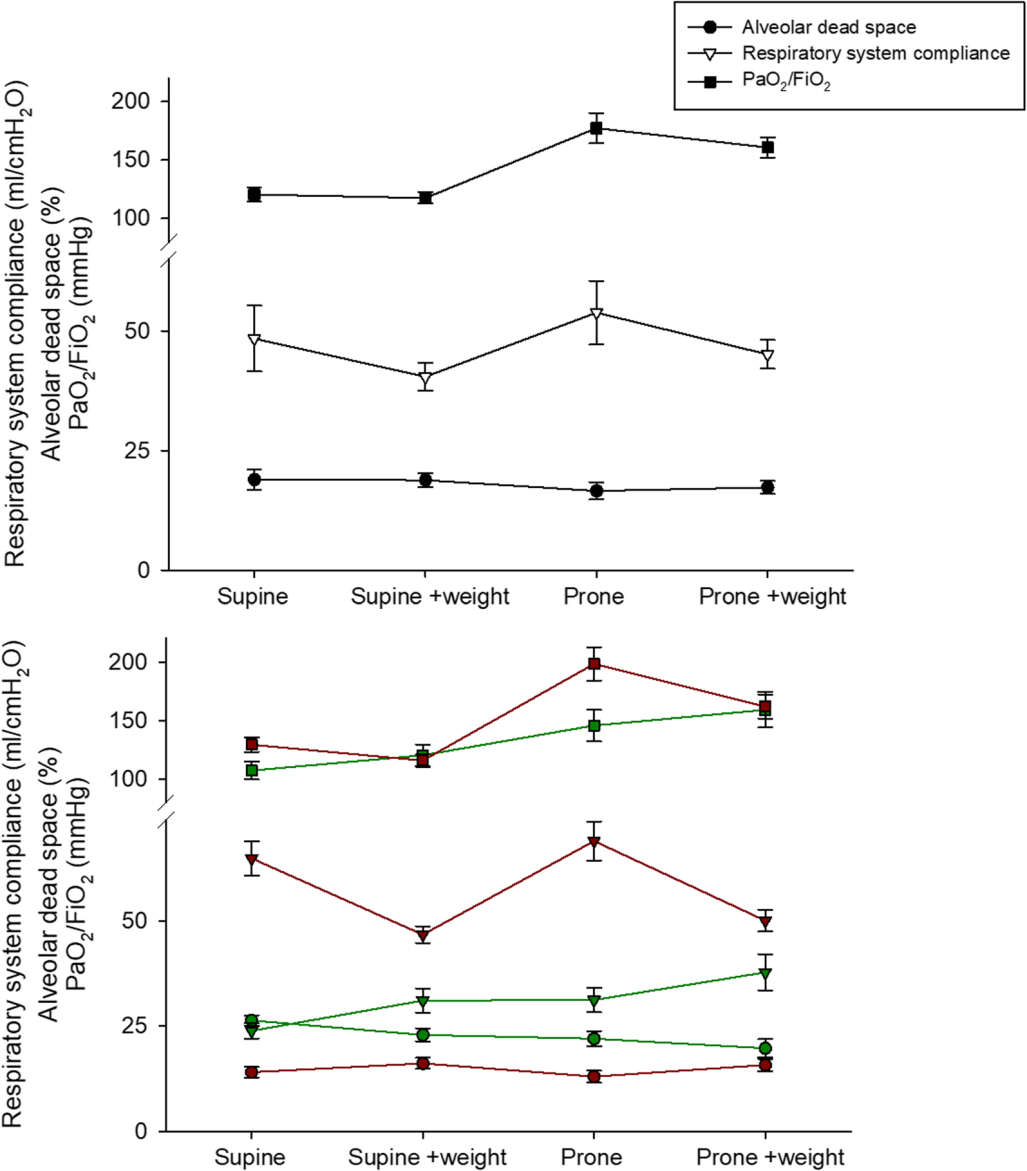
Comparison of gas exchange and mechanical properties of the respiratory system in different phases of the study. Upper panel: whole cohort. Lower panel: patients with lower (green) or higher (red) respiratory system compliance at enrolment

Table 2 and Fig. 1, upper panel shows the effects of chest wall loading on gas exchange, dead space and the mechanical properties of the respiratory system during both the supine and prone position. Briefly, neither oxygenation nor respiratory system compliance did change between supine and supine + weight; both increased during prone positioning and were unaffected by chest wall loading in the prone position. Alveolar dead space was unchanged during all the study phases.

**Table 2:** Comparison of gas exchange and mechanical properties of the respiratory system in different phases of the study

	Supine	Supine + weight	Prone	Prone + weight	*p* value
pH	7.36 ± 0.03	7.36 ± 0.02	7.37 ± 0.03	7.37 ± 0.02	0.2470
PaO_2_ (mmHg)	68.9 ± 4.6	67.8 ± 8.3	101 ± 15°*	92.4 ± 12.4°*^§^	< 0.0001
PaCO_2_ (mmHg)	48.8 ± 8.0	48.7 ± 7.0	47.8 ± 7.5	48.1 ± 6.8	0.9070
PaO_2_/FiO_2_ (mmHg)	121 ± 18	118 ± 15	177 ± 41°*	161 ± 27°*^§^	< 0.0001
EtCO_2_ (mmHg)	39.1 ± 3.4	39.3 ± 3.8	39.5 ± 3.98	39.6 ± 4.34	0.9465
Ventilatory ratio	1.72 ± 0.42	1.71 + 0.38	1.68 ± 0.40	1.69 ± 0.37	0.9701
Alveolar dead space (%)	19.0 ± 6.76	18.9 ± 4.6	16.6 ± 5.64	17.4 ± 4.4	0.1364
Airway plateau pressure (cmH_2_O)	21.3 ± 5.0	21.8 ± 2.1	19.5 ± 3.5*	20.5 ± 2.1	0.0193
Airway driving pressure (cmH_2_O)	11.9 ± 4.32	12.4 ± 2.06	10.1 ± 3.02*	11.1 ± 2.28	0.0063
Respiratory system compliance (ml/cmH_2_O)	48.5 ± 21.8	40.4 ± 9.4	53.8 ± 21.0*	45.2 ± 9.4	0.0041
Mean arterial pressure (mmHg)	78.4 ± 5.5	78.5 ± 5.5	79.6 ± 5.7	79.6 ± 5.5	0.6341
Heart rate (1/min)	77.1 ± 8.1	77.5 ± 8.3	78.3 ± 7.6	78.4 ± 7.8	0.8861

The analysis on the variables was performed by one-way analysis of variance with appropriate post hoc tests. Two-tailed *p* values < 0.05 were considered statistically significant

°*p* < 0.05 versus supine; *p < 0.05 versus supine + weight; ^§^*p* < 0.05 versus prone

### Patients with higher and lower compliance

A total of 24 patients (60%) were classified as higher respiratory system compliance at study enrolment. Table 1 shows the main characteristics at baseline, parameters at enrolment in the study and outcomes in patients with higher vs. lower compliance. Briefly, patients with higher compliance were on average younger, and there were no other differences in baseline characteristics. At enrolment, patients with higher compliance had been in the ICU for a shorter time (8.1 ± 2.9 vs. 10.3 ± 1.1 days, *p* = 0.0061). On the day of enrolment, patients with higher compliance were ventilated with a lower PEEP (8.8 ± 1.0 vs. 10.3 ± 1.9 cmH_2_O, *p* = 0.0024) and a lower FiO_2_ (0.54 ± 0.06 vs. 0.64 ± 0.07, *p* < 0.0001), a lower respiratory rate (15.8 ± 1.2 vs. 22.6 ± 2.0 1/min, *p* < 0.0001) and a higher tidal volume (8.0 ± 0.8 vs. 6.2 ± 1.1 ml/kg PBW, *p* < 0.0001), while minute ventilation was not significantly different. Despite a similar ICU and hospital length of stay, ICU mortality was significantly lower in patients with higher compliance.

### Effects of chest wall loading by compliance

Table 3 and Fig. 1, lower panel shows the effects of chest wall loading on gas exchange, dead space and the mechanical properties of the respiratory system during both the supine and prone position in patients with higher and lower respiratory system compliance. In patients with higher compliance, oxygenation did not change between supine and supine + weight, increased with prone positioning and did not change with prone positioning + weight; alveolar dead space did not change in any of the steps of the study; respiratory system compliance was significantly reduced from supine to supine + weight, increased with prone positioning and decreased again with prone + weight. On the other side, in patients with lower compliance, oxygenation significantly increased from supine to supine + weight, and it further increased with prone and prone + weight; alveolar dead space decreased from both supine and prone position after chest wall loading, and respiratory system compliance significantly increased from supine to supine + weight and from prone to prone + weight.±

**Table 3:** Comparison of gas exchange and mechanical properties of the respiratory system in different phases of the study in patients with lower or higher respiratory system compliance at enrolment

	Lower Crs *N* = 16	Higher Crs *N* = 24	P _Crs_	P _Phase_	P _Crs*Phase_
*pH*			< 0.0001	< 0.0001	< 0.0001
Supine	7.33 ± 0.02^#^	7.38 ± 0.02			
Supine + weight	7.34 ± 0.02^#^	7.38 ± 0.02			
Prone	7.34 ± 0.02^#^	7.39 ± 0.02			
Prone + weight	7.35 ± 0.02°^#^	7.39 ± 0.02			
*PaO*_*2*_* (mmHg)*			0.0425	< 0.0001	< 0.0001
Supine	67.9 ± 5.5	69.6 ± 3.8			
Supine + weight	76.1 ± 6.8°^#^	62.3 ± 2.4			
Prone	91.8 ± 9.4°*^#^	107 ± 15.2°*			
Prone + weight	100 ± 12.2°*^§#^	87.1 ± 9.5°*			
*PaCO*_*2*_* (mmHg)*			< 0.0001	< 0.0001	< 0.0001
Supine	57.6 ± 4.10^#^	43.0 ± 2.88			
Supine + weight	56 ± 4.20^#^	43.8 ± 2.98			
Prone	55.8 ± 4.07°^#^	42.4 ± 3.12			
Prone + weight	55 ± 4.53°^#^	43.5 ± 3.04			
*PaO*_*2*_*/FiO*_*2*_* (mmHg)*			< 0.0001	< 0.0001	< 0.0001
Supine	107 ± 15.4^#^	129 ± 15.3			
Supine + weight	120 ± 18.5°	116 ± 13.1			
Prone	146 ± 27.0°*^#^	198 ± 34.9°*			
Prone + weight	159 ± 30.4°*^§^	162 ± 25.1°*			
*EtCO*_*2*_* (mmHg)*			< 0.0001	< 0.0001	< 0.0001
Supine	42.4 ± 2.58^#^	36.9 ± 1.65			
Supine + weight	43.1 ± 2.78^#^	36.7 ± 1.58			
Prone	43.4 ± 2.92^#^	36.8 ± 1.76			
Prone + weight	44.1 ± 2.98°^#^	36.5 ± 1.61			
*Ventilatory ratio*			< 0.0001	< 0.0001	< 0.0001
Supine	2.12 ± 0.28^#^	1.45 ± 0.22			
Supine + weight	2.07 ± 0.27°^#^	1.48 ± 0.23			
Prone	2.06 ± 0.28°^#^	1.43 ± 0.23			
Prone + weight	2.03 ± 0.27°*^§#^	1.47 ± 0.22			
*Alveolar dead space (%)*			< 0.0001	< 0.0001	< 0.0001
Supine	26.4 ± 2.45^#^	14.1 ± 3.13			
Supine + weight	22.9 ± 3.10°^#^	16.2 ± 3.27			
Prone	21.9 ± 3.51°^#^	16.9 ± 1.14			
Prone + weight	19.8 ± 4.34°*^§#^	15.8 ± 3.68			
*Airway plateau pressure (cmH*_*2*_*O)*			< 0.0001	< 0.0001	< 0.0001
Supine	27.1 ± 1.8^#^	17.4 ± 1.2			
Supine + weight	23.4 ± 2.2°^#^	20.6 ± 1.1°			
Prone	23.3 ± 2.1°^#^	16.9 ± 1.1*			
Prone + weight	21.4 ± 2.8°*^§#^	19.9 ± 1.1^§^			
*Airway driving pressure (cmH*_*2*_*O)*			< 0.0001	< 0.0001	< 0.0001
Supine	16.8 ± 1.8^#^	8.67 ± 1.3			
Supine + weight	13.2 ± 2.4°^#^	11.9 ± 1.0°			
Prone	13.1 ± 2.4°^#^	8.17 ± 1.2*			
Prone + weight	11.1 ± 3.3°*^§#^	11.1 ± 1.2^§^			
*Respiratory system compliance (ml/cmH*_*2*_*O)*			< 0.0001	< 0.0001	< 0.0001
Supine	23.9 ± 3.56^#^	64.8 ± 10.0			
Supine + weight	30.9 ± 5.7°^#^	46.8 ± 4.8°			
Prone	31.1 ± 5.7°^#^	68.9 ± 11.4*			
Prone + weight	37.8 ± 8.7°*^§#^	50.0 ± 6.2^§^			

Crs: respiratory system compliance

The analysis was performed by factorial analysis of variance for repeated measurements, with the phase of the study as a within-subject factor, and the lower or higher respiratory system compliance at enrolment as a fixed, between-subject factor. The interaction effect between respiratory system compliance on the phase of the study was included in the model. The statistical significance of the within-subject factors was corrected with the Greenhouse–Geisser method. In the case of statistically significant interactions, pairwise post hoc multiple interaction comparisons have been carried out, according to Tukey honestly significant difference method for multiple comparison. Adjusted *p* values are reported where appropriate and are expressed as the statistical significance of the between-group comparison (*P*_Crs_), the statistical significance of the within-group comparison (*P*_Phase_) and the statistical significance of the interaction between baseline compliance and the phase of the study (*P*_Crs*Phase_). Two-tailed *p* values < 0.05 were considered statistically significant

°*p* < 0.05 versus supine; *p < 0.05 versus supine + weight; ^§^*p* < 0.05 versus prone; ^#^*p* < 0.05 versus normal Crs

**Fig. 2:**
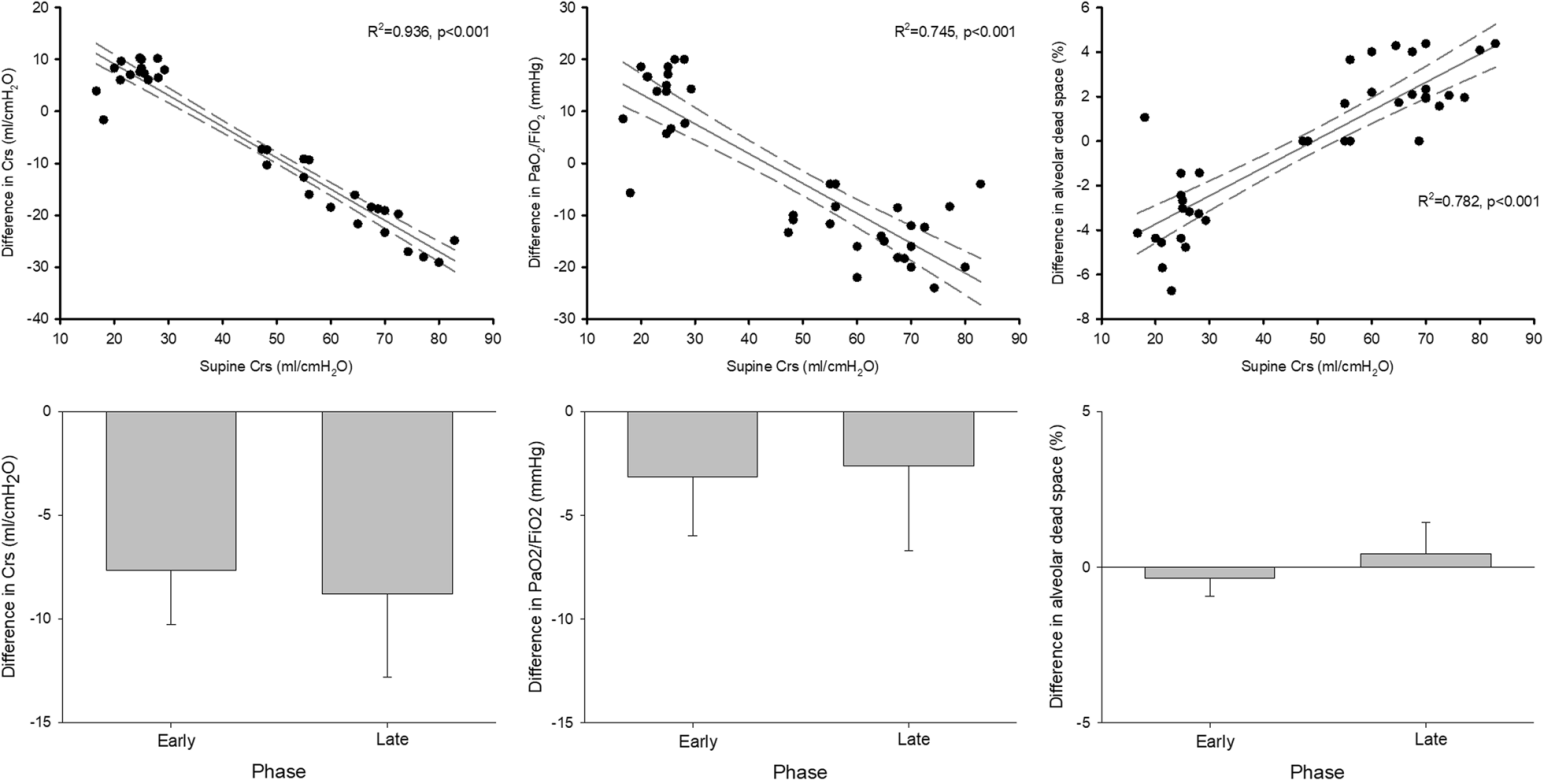
UPPER: correlation between the supine respiratory system compliance and the difference in respiratory system compliance (left panel), PaO_2_/FiO_2_ (middle panel) and alveolar dead space (right panel) between supine and supine + weight. LOWER: comparison of the difference in respiratory system compliance (left panel), PaO_2_/FiO_2_ (middle panel) and alveolar dead space (right panel) between supine and supine + weight in patients enrolled in the early or late phase of the disease

Figure 2, upper panel shows a statistically significant linear correlation between the respiratory system compliance in the supine position at enrolment in the study and the change in respiratory system compliance, PaO_2_/FiO_2_ and alveolar dead space after loading the chest wall in the supine position.

The effect of chest wall loading on respiratory system compliance, PaO_2_/FiO_2_ and alveolar dead space was not statistically different between patients enrolled in the early or late phase of C-ARDS (Fig. 2, lower panel).

Additional file 2: Figure S2 shows the statistically significant linear correlation between the airway driving pressure in the supine position at enrolment in the study and the change in respiratory system compliance (R^2^ = 0.840, *p* < 0.001), PaO_2_/FiO_2_ (R^2^ = 0.6830, *p* < 0.001) and alveolar dead space after loading the chest wall in the supine position (*R*^2^ = 0.693, *p* < 0.001).

## Discussion

To the best of our knowledge, this is the first cohort of C-ARDS patients in which the effect of chest wall loading was assessed both in the supine and prone position. The main findings of the current investigation are that: (1) chest wall loading did not change respiratory system compliance, gas exchange or alveolar dead space in an unselected cohort of critically ill patients with C-ARDS; (2) the effect of chest wall loading was modulated by the respiratory system compliance at enrolment in the study, so that patients with a lower compliance experienced an improvement in gas exchange, dead space and the mechanical characteristics of their respiratory system; (3) the lower the respiratory system compliance at enrolment, the greater was the improvement, while the phase of disease is not associated with the response. Even a 6-h period of chest wall loading was safe, as no signs of discomfort or distress were recorded.

In general, respiratory system mechanics depend on the elastic properties of lung and chest wall. The regional distensibility of the chest wall, which is composed by the rib cage and abdomen, varies markedly from site to site, with dorsal regions being more rigid than ventral ones, and the rib cage being less flexible than the abdomen [27]. Prone positioning is a manoeuver used in patients with moderate-severe ARDS to improve oxygenation and reduce mortality [3]. The change to prone position reversibly stiffens the relatively compliant anterior portions of the chest wall and ventral abdomen, relieves the superimposed pressure of both the heart and the abdomen on the lungs and induces a more uniform distribution of tidal volume by reversing the vertical pleural pressure gradient [2, 28]. Since pulmonary perfusion remains preferentially distributed to the dorsal regions, an overall improved alveolar ventilation/perfusion matching also occurs.

If the restricting effect on the anterior chest wall is considered the main pathophysiologic correlate of prone positioning, then at least some of its effects should be replicated by stiffening the chest surface. Chest wall restriction has long been used as a model for studying the physiology of restrictive diseases, respiratory muscle weakness, and the effects of general anesthesia and muscle relaxants [29–31]. Indeed, external chest wall compression uniformly reduces chest wall compliance; if tidal volume and PEEP do not change, an increased driving pressure and a reduced respiratory system compliance are expected [9]. However, this can only occur provided that lung compliance does not simultaneously improve by the imposed stiffening of the chest wall.

Before the COVID-19 era, only few papers investigated the effects of chest wall loading in critically-ill patients. In 11 supine, mechanically ventilated patients with acute lung injury, loading of the anterior chest wall with a 10 kg sand bag led to a 25% decrease in EELV and an increase in compliance. While oxygenation did not change in the whole cohort, patients who improved their oxygenation were the same who reduced their chest wall compliance [10]. Samanta et al. report two cases of trauma where prone position could not be performed, chest wall compression with 2-kg weight on each side of the chest wall bilaterally while the patients were in the supine position led to significant improvements in oxygenation [32]. Notably, several recent reports of C-ARDS patients, mainly enrolled in a late phase of their illness, described a paradox, unexpected improvement in respiratory system compliance and gas exchange in response to anterior chest wall loading [11–16].

In an unselected sample of C-ARDS patients, we were unable to find any effect of chest wall loading on respiratory system compliance, gas exchange or dead space during either supine or prone position. In fact, previous reports [11–16] found that the paradoxical increase in respiratory system compliance with anterior chest compression was mostly due to the decrease in overdistension because of the decrease in end-expiratory lung volume. In the present study, indeed, patients who increased Crs during chest wall compression had higher baseline PEEP, plateau and driving pressure. It is interesting to note that a positive effect of chest wall loading on respiratory mechanics and oxygenation was only seen in patients with signs of overdistension. To test this hypothesis, we analyzed the effects of chest wall compression depending on the basal driving pressure, considered a surrogate for lung strain [25]. We found a statistically significant, linear effect of baseline airway driving pressure and the response to chest wall loading, so that the higher the strain, the higher was the improvement in compliance and oxygenation, and the higher was the reduction in alveolar dead-space.

The apparent inconsistency between our results and the improvements seen in previous reports might lie in the fact that all the patients included in those reports had a severely reduced respiratory system compliance, ranging from 13 to 35 ml/cmH_2_O [11–16]. Similar to those findings, we also noticed a positive effect of chest wall loading in terms of gas exchange, mechanical characteristics and a reduction in alveolar dead space when considering only patients with a reduced supine respiratory system compliance, whereas in patients with a higher respiratory system compliance oxygenation improved only with prone positioning with no effects of chest wall loading.

On the other side, prone positioning led to an improvement in oxygenation, respiratory system compliance and alveolar dead space in the whole cohort, irrespective of the baseline degree of distention or the value of respiratory system compliance. This is in line with the available literature of both COVID-19 and non-COVID-19 patients [3, 33, 34] and confirms how prone positioning is the standard of care in patients with moderate-severe forms of ARDS [35, 36]. Notably, the response to chest wall loading in the supine position was not able to predict the physiologic response to prone positioning: such manoeuver should better be used as a way to assess whether the patient is overdistended at end-inspiration than as a proxy of the response to prone positioning, and be used to optimize PEEP, tidal volume or both, rather than to decide whether to proceed with prone positioning.

That prone positioning led to improved respiratory system compliance while anterior chest wall loading did not is a finding that deserves some discussion. Chest wall loading increases intrapleural pressure, and this should normally lead to a proportional rise in airway plateau pressure and hence to an increased driving pressure and a reduced compliance. However, if the aerated lung volume is reduced to a very low extent, the remaining lung units operate closer to their non-compliant upper range [8]. In such cases, chest wall loading leads to reduction in the distension of previously overstretched lung units [13], allowing them to operate on a more linear portion of their pressure–volume curves [12]. As a consequence, respiratory system compliance can only improve if a significant amount of lung units were overdistended right before chest wall loading. On the other side, prone positioning does more than selectively stiffening the relatively compliant anterior chest wall: it relieves the lungs from the weight of the heart and reduced the cephalad push of abdominal pressure on dorsal lung areas [2].

Based on the generally reduced respiratory system compliance of the reports available in the literature on chest wall loading, we hypothesized that the effect of such manoeuver could in fact depend upon the baseline level of respiratory system compliance. In our case-mix, 16 out of 40 patients (40%) had a low respiratory system compliance at enrolment in the study, which is in line with other studies [23]. Patients in the lower compliance group were on average older, had a lower absolute body weight and a similar pattern of comorbidities as compared with patients with higher compliance; the two groups had a similar severity at ICU admission, despite those with reduced compliance had a higher mortality. Upon enrolment, patients in the reduced compliance group were ventilated with a lower tidal volume, a higher respiratory rate and a higher PEEP. With chest wall loading, respiratory system compliance increased in this group by 30.5%, while it decreased by 26% in the higher compliance group. This finding suggests that in those patients with reduced baseline compliance, some degree of end-tidal overinflation occurred within the aerated part of the diseased lung; chest wall loading then leads to a reduction in the end-expiratory lung volume, while at the same time easing the end-inspiratory lung overdistension sufficiently to offset the reduction in chest wall compliance, causing a downward shift of the pressure volume curve, with reduction in tidal hyper-inflation and possibly increase in tidal recruitment [15, 16]. Since tidal volume was unchanged, such improvement in compliance in patients with lower respiratory system compliance implies recruitment to a higher lung volume. This is notable, as both groups of patients had PEEP titrated to the best respiratory system compliance. Indeed, the main limitation to the titration of PEEP to respiratory system mechanics is that, given the high degree of inhomogeneity in the lungs of ARDS patients, any change in PEEP introduces regional lung overdistension and recruitment at the same time, making assumptions on the effect of PEEP on the lung volume recruited unreliable. As a matter of fact, it has been shown how PEEP selection with lung mechanics-based methods is unrelated to the lung recruitability and may lead to higher values applied to patients with lower recruitability [37]. Because of the heterogeneity of the disease, the effects of PEEP in COVID-19 patients have been shown to be highly variable and cannot be easily predicted by respiratory system characteristics [38]. This implies caution in mechanic-based methods for the selection of PEEP in COVID-19 patients.

Moreover, a further 20% improvement in compliance was found when chest wall loading was applied in patients in the prone position, suggesting a reduction in hyperinflation in the dorsal lung region despite the already compressed anterior chest wall of prone positioning [14].

The effects of chest wall loading on the mechanical characteristics of the respiratory system and gas exchange are considered to depend on a reduction in lung overinflation. Indeed, we cannot exclude that patients with a reduced respiratory system compliance are the same patients in which an inadequate setting of the mechanical ventilator leads to some degree of overinflation; notably, patients in the low compliance group also had a statistically significant higher PEEP, which was shown to be associated with a larger extent of tidal and maximal hyperinflation in patients with pulmonary ARDS [39].

Another finding consistently reported with a positive response to chest wall loading in the available literature is the association with a late phase of the disease [11, 12, 14, 16]. This has been interpreted as patients in the late phase are more overdistended, as unresolving C-ARDS may be characterized by impressive loss of aeratable lung units, in part due to fibroblastic proliferation and organization within the parenchyma [8]. We enrolled patients across a wide range of days from ICU admission and classified patients into early (within the first week) and late phase. We were unable to find any association between the effect of chest wall loading and the early and late phase of C-ARDS. Indeed, studies from non-COVID-19 ARDS have shown that the persistent phase of ARDS for 7-days was not associated with any change in respiratory mechanics or oxygenation [40].

Several limitations need to be considered when interpreting our findings. The results are not sufficient to clearly identify the underlying mechanisms, as we did not assess lung volumes, regional ventilation distribution or partitioned the mechanical characteristics of the chest wall and the lung. The lack of esophageal pressure monitoring significantly lessens the interpretation of our findings. Moreover, a single weight was used for all patients, rather than individualizing the effect of chest wall loading; in particular, we arbitrarily chose 10 kg because we previously noted that the pressure exerted by this weight seemed to induce significant changes to the respiratory system [14]. However, we acknowledge that it is unclear, from the available literature, which is the most appropriate weight to be applied. Rezoagli et al. applied a 5 kg weight [13] while Carteaux et al. [11] applied a saline bag which generated a pressure of 80 cmH_2_O over the chest. Kummer et al. [12] performed a manual compression without quantifying the weight in terms of kilograms. We think that this issue still needs further explorations, and ideally the weight might be patient-tailored.

Similarly, the duration of chest wall loading sessions was standardized and arbitrarily defined. Again, the literature lacks information as to the ideal duration of any such session. We aimed to assess the effect of chest wall loading in the setting of the need for prone positioning, which is known to be associated with improvements in the clinical outcome. Since international guidelines recommend that patients with moderate–severe ARDS receive prone positioning for at least 12 h per day [36], we designed a study in which a 12-h session of prone positioning was combined with chest wall loading, hence the 6-h periods. The small sample size does not allow generalizability to patients with different body morphologies, positions, or illnesses. Eventually, any benefit of long-term chest wall loading is not proved, and its impact on gas exchange remains unclear.

## Conclusions

In conclusion, while prone positioning led to improved oxygenation and mechanics in all patients, chest wall loading had no effects on respiratory system compliance, gas exchange or alveolar dead space in an unselected cohort of critically ill patients with C-ARDS. Moreover, chest wall loading did not predict the response to prone positioning. Only patients with a low respiratory system compliance experienced an improvement, with a higher response the lower the baseline compliance. Further studies will be required to identify the optimal timing, duration and weight of chest wall loading, as well as any impact on patient-centered outcomes. In the meantime, we suggest to perform chest loading in all patients suffering from ARDS and with a reduced respiratory system compliance, to check for unexpected improvements in compliance which should prompt consideration of modifying the ventilator settings and to consider such manoeuver only in responders in conjunction with prone positioning, as this could lead to further improvements in respiratory mechanics and gas exchange.

## Supplementary Information

**Additional file 1**. **Supplementary Figure S1.** Scheme of the study protocol.

**Additional file 2**. **Supplementary Figure S2.** Correlation between the supine airway driving pressure and the difference in respiratory system compliance (left panel), PaO_2_/FiO_2_ (middle panel) and alveolar dead space (right panel) between supine and supine + weight.

## Data Availability

The datasets used and/or analyzed during the current study are available from the corresponding author on reasonable request.
