# Supplementary material for: Chest wall loading during supine and prone position in patients with COVID-19 ARDS: effects on respiratory mechanics and gas exchange
Source: Crit Care. 2022 Sep 13;26:277. doi: 10.1186/s13054-022-04141-7 (PMC9470071; doi:10.1186/s13054-022-04141-7)
Supplement: Supplementary file 1 — Additional file 1. Supplementary Figure S1. Scheme of the study protocol. [file 13054_2022_4141_MOESM1_ESM.docx]

**Supplementary Figure S1.** Scheme of the study protocol.


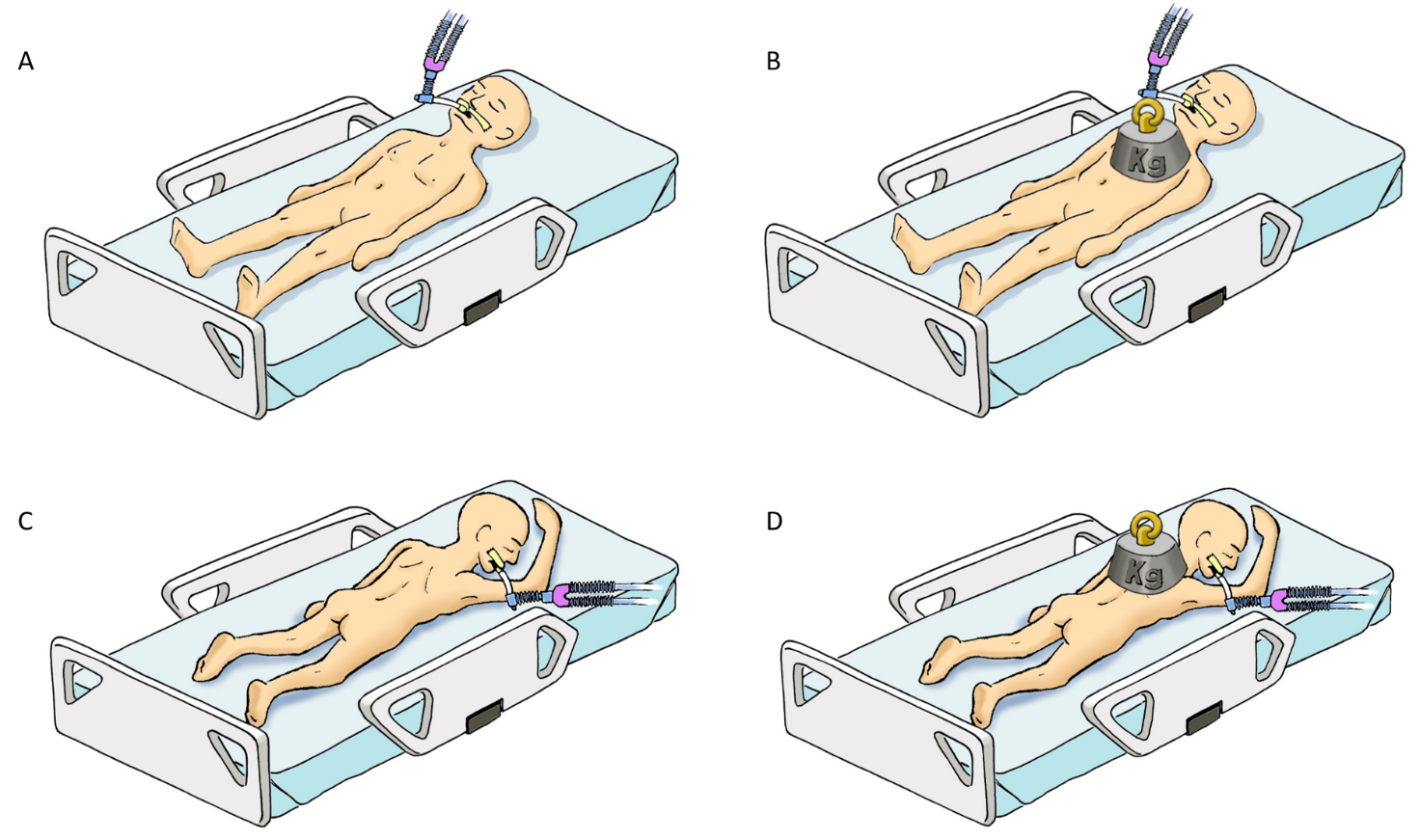


A: Supine position; B: Supine +weight; C: Prone position; D: Prone +weight
