# Supplementary material for: Chest wall loading during supine and prone position in patients with COVID-19 ARDS: effects on respiratory mechanics and gas exchange
Source: Crit Care. 2022 Sep 13;26:277. doi: 10.1186/s13054-022-04141-7 (PMC9470071; doi:10.1186/s13054-022-04141-7)

**Supplementary Figure S2.** Correlation between the supine airway driving pressure and the difference in respiratory system compliance (left panel), PaO_2_/FiO_2_ (middle panel) and alveolar dead space (right panel) between supine and supine +weight.


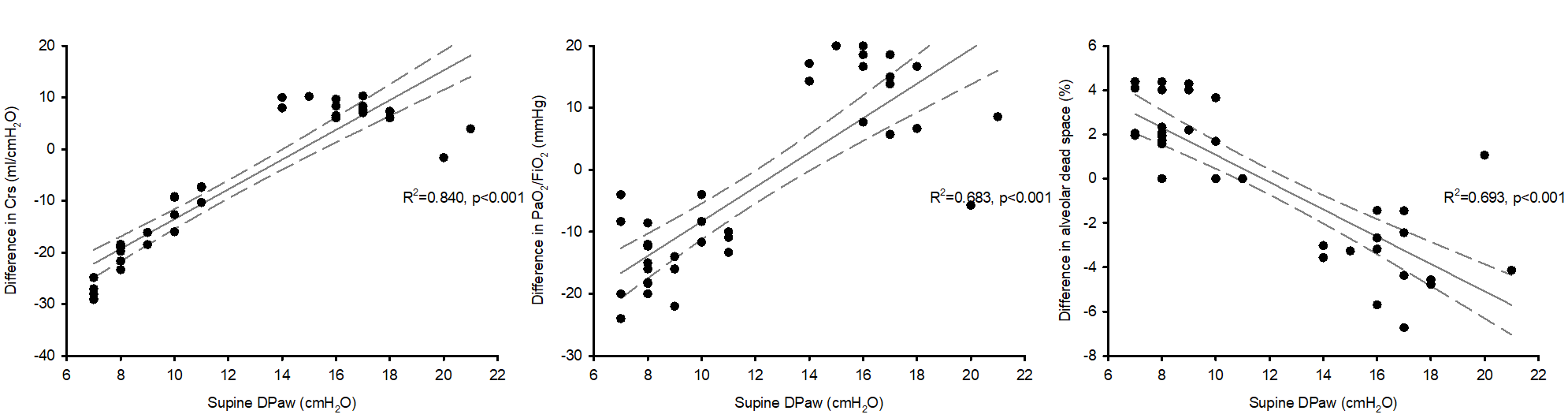

Supplement: Supplementary file 2 — Additional file 2. Supplementary Figure S2. Correlation between the supine airway driving pressure and the difference in respiratory system compliance (left panel), PaO2/FiO2 (middle panel) and alveolar dead space (right panel) between supine and supine + weight. [file 13054_2022_4141_MOESM2_ESM.docx]
